# Traditional Chinese Medicine Injections in the Treatment of Diabetic Foot: A Systematic Review and Meta-Analysis

**DOI:** 10.1155/2018/4730896

**Published:** 2018-10-08

**Authors:** Lizi Tan, Qingyang Shi, Chunxiang Liu, Junhua Zhang, Hui Wang, Jingbo Zhai

**Affiliations:** ^1^Tianjin University of Traditional Chinese Medicine, Tianjin 300193, China; ^2^Evidence-based Medicine Center of Tianjin University of Traditional Chinese Medicine, Tianjin 300193, China

## Abstract

**Context:**

The role of traditional Chinese medicine injections (TCMIs) in diabetic foot (DF) has not been well estimated.

**Objective:**

To evaluate the clinical effective rate, safety, and the financial cost of TCMIs in treating DF and ulcer wound healing.

**Methods:**

We searched PubMed, Embase, CENTRAL, China National Knowledge Infrastructure (CNKI), VIP database, and Wanfang database from inception to May 2018 to find all randomized control trials (RCTs) related to TCMIs in DF treatment. The search items were “Traditional Chinese Medicine Injection” AND “Diabetic foot or Diabetic foot ulcer” AND “random”.

**Study Selection and Synthesis:**

Only RCTs of TCMIs combined conventional therapies versus conventional therapies and that can be quantitatively synthesized were included. Finally, 17 studies and 1294 participants were included after extraction. Two investigators independently extracted and analyzed the data using RevMan5.3 software.

**Results:**

The overall clinical effective rate of TCMI groups is higher than that of control groups [RR=1.27, 95CI % (1.20, 1.34), P<0.00001] based on fixed effect model analysis. Regarding motor nerve conduction velocity of median nerve and peroneal nerve, TCMI group showed a significant improvement (MD=3.84[2.28, 5.41], P<0.00001; MD=2.89[0.63, 5.15], P=0.01). Regarding plasma viscosity TCMI group showed a statistically difference (MD=0.27[0.04, 0.49], P=0.02). In terms of blood viscosity at high shear rate, there was an improvement of TCMI group (MD=0.36[0.05, 0.67], P=0.02). However, sensory nerve conduction velocity of peroneal nerve and median nerve showed a contradiction to motor nerve conduction velocity, respectively (MD=2.59[-1.69, 6.87], p=0.24; MD=2.73[-0.96, 6.43], P=0.15).

**Conclusion:**

The data of this study shows that TCMIs can bring benefits to patients with diabetic foot. However, due to low methodological quality of included RCTs, more rigorous designed RCTs with large sample size are recommended to provide more high-quality evidence.

## 1. Introduction

Diabetic foot (DF) is the infection, ulceration, or destruction of tissues of the foot associated with neuropathy and/or peripheral vascular disease (PVD) in the lower extremity of people with diabetes [1]. Diabetic peripheral neuropathy (DPN) and microangiopathy are the most significant risk factors for DF [2]. It is one of the most severe and costly chronic complications of diabetes mellitus (DM) [3]. People with diabetes with foot ulcers experience health expenditures five times higher than those without foot ulcers [4]. It always develops from mild or moderate neural symptoms into diabetic foot ulcers (DFU) on lower extremities even leading to amputations. The amputation rate population with DM is ten to twenty times more than the nondiabetic population [5]. And there is also a twofold risk of mortality for DM population with a history of DFUs compared to those without DM [6].

The prevalence of foot ulcers of people having diabetes mellitus is 4% to 10%, and the annual population-based incidence is 1.0% to 4.1% [7, 8]. The lifetime incidence of DM people having DFU could be as high as 25% [9]. Given the rapid growth of DM population which will increase by 48% in 2045 compared to the number of 425 million [4], we are facing a rapid growing of DF patients in the following 30 years.

However, the DFU is preventable and a timely treatment for ulcers can help in the reduction of severe outcomes. A comprehensive intervention including DFU risk assessments, foot care based on prevention, education for patients and their healthcare attendants, and a multidisciplinary treating approach will lower foot complications and amputations by 85% at most [4].

Being a widely practiced and long-time-used healthcare method, traditional Chinese Medicine plays a significant role in treating DM and glycaemic control [10–15]. According to IDF DIABETES ATLAS 8th edition, intensive glycaemic control is the primary preventive method of DFU and associated with a lower risk of amputation and sensory numbness [4, 16]. Traditional Chinese medicine injection (TCMI) is sterile liquid of active ingredients that extracted from the natural drugs. The TCMIs with the clinical efficacy of promoting blood circulation to remove blood stasis are now being widely used in China on preventing and treating of DPN and they are subsequent [17, 18]. However, there was no sufficient evidence-based medicine (EBM) support of that for clinicians and specialists. We performed this systematic review and meta-analysis to investigate the clinical efficacy and safety for TCMIs on DFU.

## 2. Methods

We strictly followed the instruction of Preferred Reporting Items for systematic reviews and meta-analyses: the PRISMA statement during the process of this review [19].

### 2.1. Inclusion and Exclusion Criteria

We included all the randomized controlled trials (RCTs) applying TCMIs in the treatment of DFU in patients with DM. Participants are diagnosed as diabetic foot and there are no restrictions of age, gender, and course of disease. TCMIs are the injections extracted from herbs, single or mixture herbal formulas. Interventions in trial group are one kind of TCMI with basic care or this TCMI combined conventional therapies with basic care. The basic care and conventional therapies should remain the same in the control group in the same RCT.

Exclusion criteria were as follows: ① duplicates; ② systematic reviews and/or meta-analyses; ③ catalogue, indexes, and conferences; ④ irrelevant topics; ⑤ RCTs using more than one traditional Chinese medicine injection; and ⑥ studies that cannot be quantitative synthesized.

There were no limits on publication status or language.

### 2.2. Search Strategy

PubMed, Embase, CENTRAL, China National Knowledge Infrastructure (CNKI), VIP Database for Chinese Technical Periodicals (VIP), and Wanfang databases were searched from inception to May 2018. The search items were “Traditional Chinese Medicine Injection or Zhong Yao Zhu She Ye or Zhu She Ye” AND “Diabetic foot or Diabetic foot ulcer or Tang Niao Bing Zu or Tang Niao Bing Zu Kui Yang” AND “random”.

### 2.3. Data Extraction

Two reviewers screened and extracted the basic information independently by using a standardized data extraction form of our own and a cross check had been made after the extraction. Disagreements were resolved by discussion and we attempted to contact the authors for the missing data. We used Zotero5.0 software to manage the bibliographies. The information we filled into the form included the following:

- General information: title, authors' names, journal, publish date, etc.
- Characteristics of the RCTs: sample size, age, gender, course of disease, interventions, etc.
- Outcomes
- Adverse reactions

### 2.4. Types of Outcome Measures

*Primary Outcomes*. Clinical effective rates are as follows.

Clinical efficacy was defined as one or more Wagner score reductions after treatment.

*Secondary Outcomes*. Nerve conduction velocity includes motor nerve conduction velocity (MCV) and sensory nerve conduction velocity (SCV).

Hemorheology includes blood viscosity and plasma viscosity.

### 2.5. Risk of Bias

Two reviewers made the assessment following the Cochrane Handbook for Systematic Reviews of Interventions 5.1.0 and the systematic review of the methodological quality assessment tools [20, 21].

- Random sequence generation (selection bias)
- Allocation concealment (selection bias)
- Blinding of participants and personnel (performance bias)
- Blinding of outcome assessment (detection bias)
- Incomplete outcome data (attrition bias)
- Selective reporting (reporting bias)
- Other bias

### 2.6. Data Synthesis

We conducted this meta-analysis through Revman5.3 software [22]. The categorical variables were analyzed by risk ratio (RR) and the continuous variables take the mean difference (MD) as the effect index, and they are all with 95% confidence interval (95% CI).

The heterogeneity among the included studies was analyzed using the chi-square test (the test level was *α*=0.1), and the heterogeneity was quantitatively determined using *I*^2^. If there is no heterogeneity or heterogeneity test result is P>0.1 or *I*^2^ <50%, the fixed effect model was applied for meta-analysis. Otherwise, we will further identify the sources of heterogeneity and then reanalyze after reducing the heterogeneity. If there still exists heterogeneity, we will run the analysis with random effects model. Subgroup analyses were conducted based on types of traditional Chinese medicine injections and we illustrated the publication bias of primary outcomes in funnel plot.

## 3. Results

### 3.1. Study Selection

**Figure 1:**
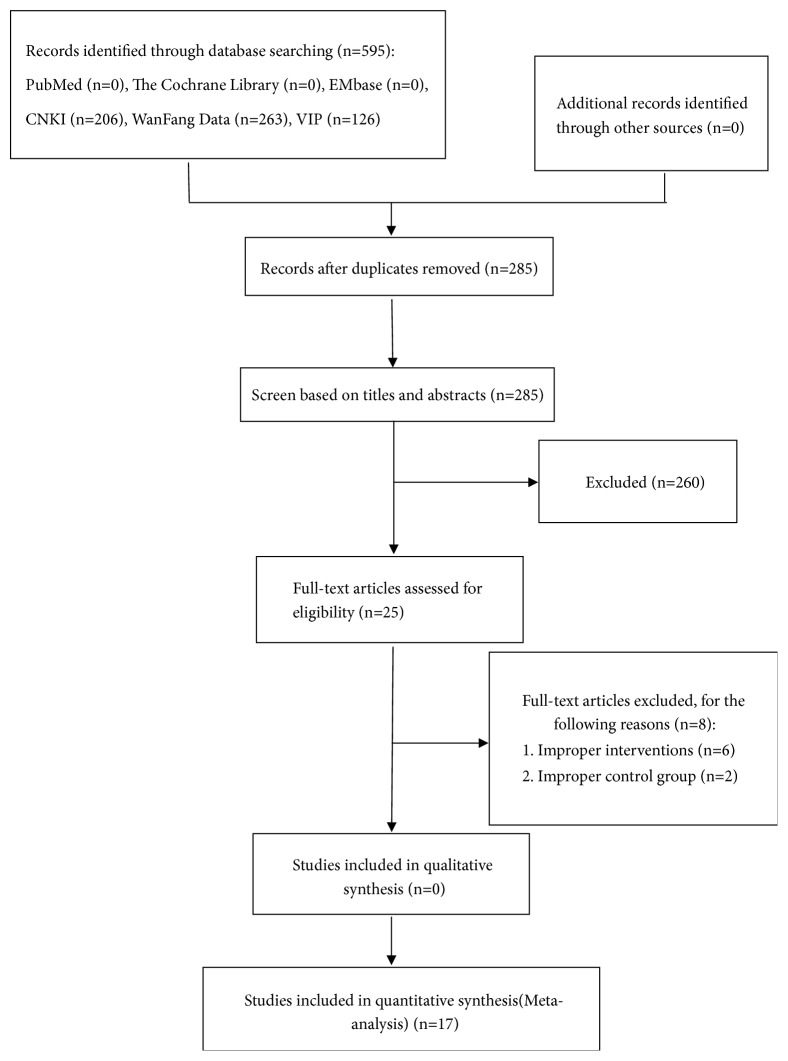
Flow diagram of study selection and identification.

We finally included 17 studies from 595 studies. The process is demonstrated in Figure 1.

### 3.2. Study Characteristics

A total of 1294 participants were included from the 17 studies [23–39]. All the data were illustrated in Tables 1 and 2 including study size, interventions, and basic information of studies.

All studies utilized Wagner scale for the classification of DFU patients when initially enrolled [40]. Among them, 13 participants were grade 0 (1%), 232 were grade 1 (17.93%), 358 were grade 2 (27.67%), 140 were grade 3 (10.82%), and 41 were grade 4 (3.17%). And 510 (39.41%) were without specific grade classification information.

### 3.3. Risk of Bias

**Figure 2:**
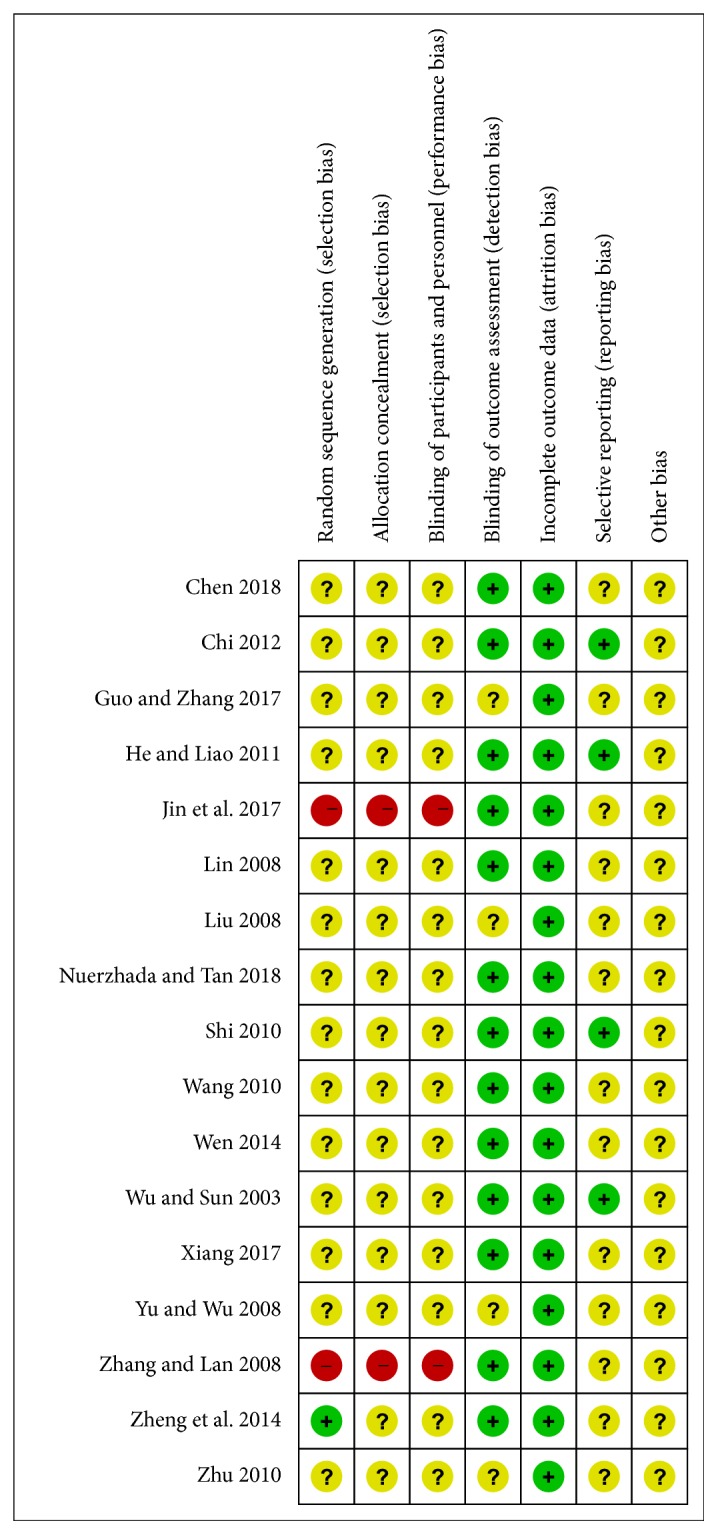
Risk of bias summary.

We used Revman5.3 software to explicitly report the methodological features for each study (Figure 2). Regarding random sequence generation, 14 studies reported “random” without specific method, 2 studies are quasi-randomized for their obvious selection bias [27, 36], and only 1 study reported using random number table [34]. Regarding blinding for patients and personnel, 2 studies had high risk and 15 studies had unclear risk. As for blinding for outcome assessment, 13 studies had low risk and 4 studies had unclear risk. Regarding incomplete outcome data, all studies had low risk of bias. Concerning selective reporting, 4 studies had low risk of bias and 13 studies had unclear risk of bias.

### 3.4. Meta-Analysis Results

#### 3.4.1. Primary Outcome: Clinical Effective Rate

**Figure 3:**
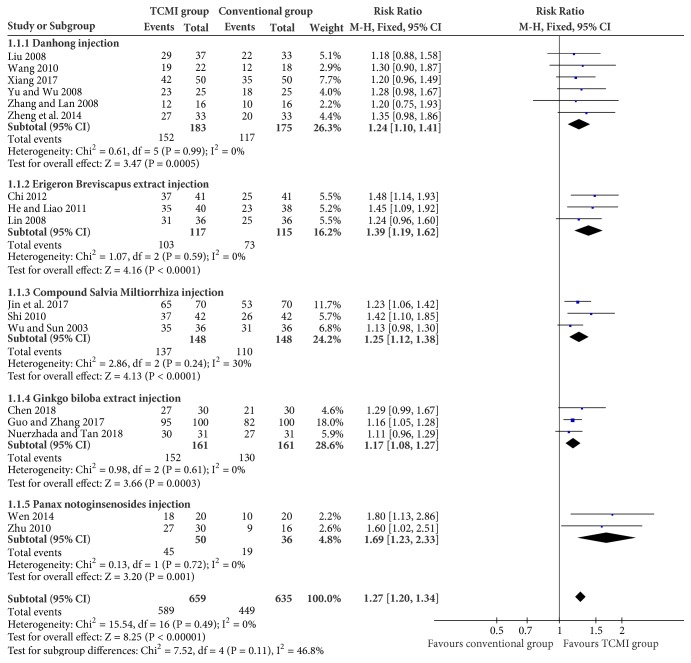
Effective rates of TCMI.

All the 17 studies and 1294 patients receiving treating were included. The overall clinical effective rate of TCMI groups is higher than that of control groups (RR=1.27, 95CI% [1.20, 1.34], P<0.00001). Analysis results of different subgroups of conventional therapies based on fixed effect model showed that all TCMI groups outperformed the conventional therapies groups (Danhong injection RR=1.24[1.10, 1.41], P=0.0005; Erigeron Breviscapus extract injection RR=1.39[1.19, 1.62], P<0.0001; Compound Salvia Miltiorrhiza injection RR=1.25[1.12, 1.38] P<0.0001; Ginkgo Biloba extract injection RR=1.17[1.08, 1.27] P=0.0003; Panax Notoginsenosides injection RR=1.69[1.23, 2.33] P=0.001) (Figure 3).

#### 3.4.2. Secondary Outcomes

All the data were analyzed on random effect model due to the heterogeneity.

**Figure 4:**
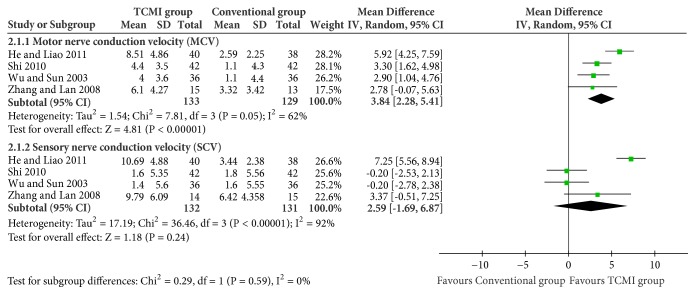
Nerve conduction velocity of median nerve.

- Nerve conduction velocity of median nerve (Figure 4)

MCV: 4 studies and 262 participants were included [23, 27, 28, 31]. TCMI group showed a significant improvement (MD=3.84[2.28, 5.41], P<0.00001).

**Figure 5:**
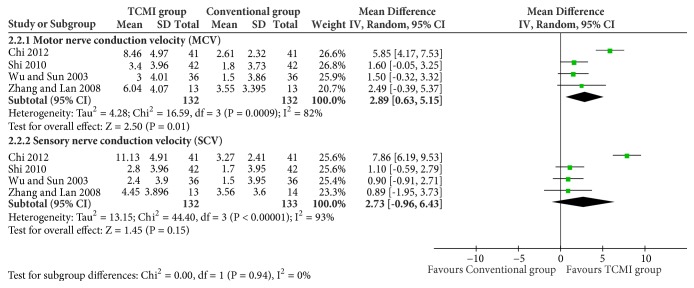
Nerve conduction velocity of peroneal nerve.

SCV: 4 studies and 263 participants were included [23, 27, 28, 31]. There was no statistical difference between two groups (MD=2.59[-1.69, 6.87], p=0.24).

(ii) Nerve conduction velocity of peroneal nerve (Figure 5)

MCV: 4 studies and 264 patients were included [23, 27, 28, 32]. TCMI group showed a statistical difference (MD=2.89[0.63, 5.15], P=0.01).

**Figure 6:**
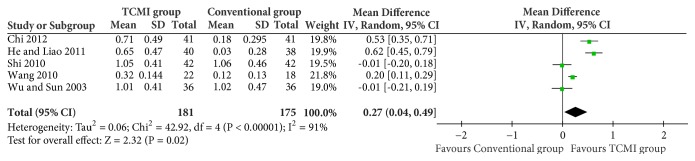
Hemorheology of plasma viscosity.

SCV: 4 studies and 265 patients were included [23, 27, 28, 32]. There was no statistical difference (MD=2.73[-0.96, 6.43], P=0.15).

(iii) Hemorheology of plasma viscosity (Figure 6)

**Figure 7:**
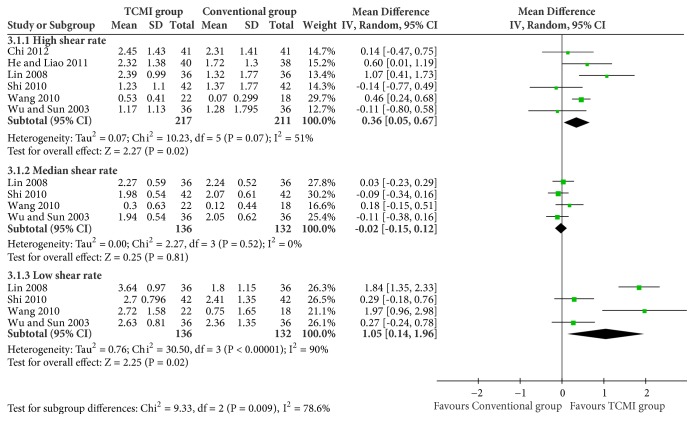
Hemorheology of blood viscosity.

A total of 5 studies and 256 participants were included [23, 28, 29, 31, 32]. It showed a statistical difference (MD=0.27[0.04, 0.49], P=0.02).

(iv) Hemorheology of blood viscosity (Figure 7)

High shear rate: 6 studies and 428 participants were included [23, 24, 28, 29, 31, 32]. There was an improvement of TCMI groups (MD=0.36[0.05, 0.67], P=0.02).

Median shear rate: 4 studies and 268 participants were included [23, 24, 28, 29]. No statistical difference existed (MD=-0.02[-0.15, 0.12], P=0.81).

Low shear rate: 4 studies and 268 participants were included [23, 24, 28, 29]. TCMI groups showed an improvement (MD=1.05[0.14, 1.96], P=0.02).

### 3.5. Adverse Events

Only 3 studies reported adverse events. Four patients had facial redness and headache in study of Chi 2012 [32]. Two patients were with rash and pruritus in study of Jin 2017 [36] and two with the same symptom in study of Xiang 2017 [37]. The adverse events were mild and disappeared afterwards, so there was no sample loss. All the other studies reported no adverse events happening.

### 3.6. Publication Bias

**Figure 8:**
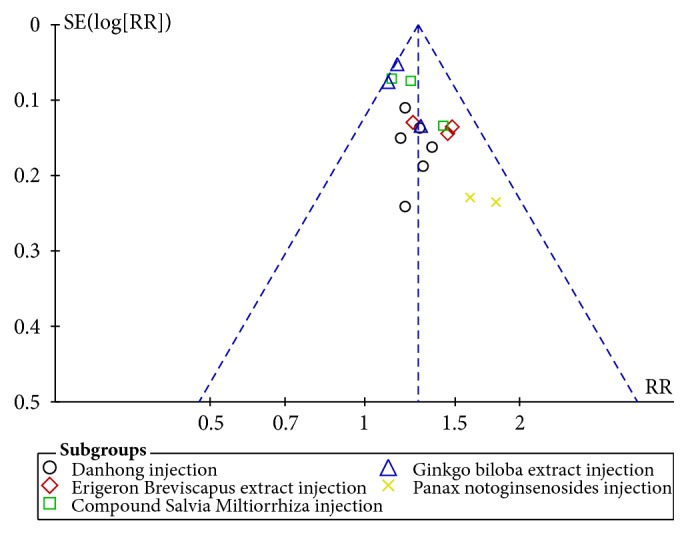
Funnel plot of the clinical effective rate.

We evaluated the possibility of publication bias by funnel plot of the clinical effective rate (Figure 8). As shown, it was generally symmetrical representing a low risk of publication bias.

## 4. Discussions

### 4.1. Summary of Main Results

We finally included 17 studies after extraction. The TCMIs they chose to use as the trail interventions concentrating on 5 different kinds are Danhong injection [25–27, 29, 34, 37], Erigeron Breviscapus extract injection (Dengzhanxixin injection) [24, 31, 32], Compound Salvia Miltiorrhiza injection (Fufang Danshen injection) [23, 28, 36], Ginkgo Biloba extract injection (Shuxuening injection) [35, 38, 39], and Panax Notoginsenosides injection (Xueshuangtong injection) [30, 33]. And we run the subgroup meta-analysis based on that.

Regarding clinical effective rate, all the five TCMI groups showed an improvement compared to conventional therapies groups no matter if it is the overall rate or subgroup rate, respectively.Using TCMIs can significantly raise the rate by 27% (P<0.00001) generally. Danhong injection was most widely used in clinic; however, evidence showed that it is not the most effective type to improve the clinical effective rate (RR=1.24[1.10, 1.41], P=0.0005). Meanwhile, the most effective type, Panax Notoginsenosides injection (RR=1.69[1.23, 2.33] P=0.001), is being used the least. Therefore, more qualified clinical trials and further researches need to be done.

Regarding the secondary outcomes, evidence suggested an improvement of TCMI groups in reducing the plasma viscosity and blood viscosity of high shear rate and low shear rate. And our evidence also supported an improvement of MCV of median and peroneal nerve, whereas no evidence supported the improvement of blood viscosity of median shear rate and SCV of both nerves. Given this contradiction, we consider a further analysis based on more qualified RCTs would help.

### 4.2. Strength and Limitations

We included 17 studies and 1294 participants totally. No sample loss happened, and all the outcomes were integrally reported at last. Regarding blinding for outcome assessors, 13 studies were evaluated with low risk of publication bias for they measured objective laboratory indexes. Also, test for subgroup difference showed no statistical differences (P=0.11, *I*^2^=46.8%). With no heterogeneity (*I*^2^=0%, P=0.49) in the analysis of overall clinical effective rate and a low heterogeneity (the largest *I*^2^=30%, P=0.24) in subgroups, we considered the internal validity moderate.

All participants are enrolled from different regions of mainland China with a balance gender ratio and most of them are middle-aged and elderly people. Within the 17 studies, only 1 reported the random number table and 14 mentioned “random” without the specific approach. Furthermore, 2 are quasi-randomized with an obvious selection bias. None of them mentioned the allocation concealment and the two quasi-randomized trial cannot conceal its allocation. That indicates a high risk of allocation bias. Besides, no participants included are classified into grade 5 in Wagner scale. Thus, we only recommend the TCMI interventions to clinicians in treating the middle-aged and elderly patients with a mild to moderate DFU classification (with a Wagner scale lower than grade 5).

Few adverse events happened in all the studies and the events happened are mild to moderate degree which will disappear after some resting. And the cost of TCMI is cheap, because most of them are in the Chinese national medical insurance list (Danhong injection, Erigeron Breviscapus extract injection, Ginkgo Biloba extract injection, Panax Notoginsenosides injection) which means 80% of the expense is covered [41]. According to the course of treatment reported, mostly 28 days, the total cost will be no more than 436.24 CNY. Compared to the significant improvement of clinical effective rate as 27%, TCMIs will only increase the average cost for an ulcer episode by 1.5% [42]. It can be considered as a cost-effective and safe strategy with a low treatment expense increase.

Although the heterogeneity of primary outcome is low, there may exist potential bias. The courses of disease were inconsistent (Table 2) and the conduction of basic care may differ from practitioners such as debridement and dressing change.

More qualified RCTs need to be included to explain the high heterogeneity in the meta-analysis of secondary outcomes. And due to a contradictory result of secondary outcomes, we hereby recommend more qualified RCTs with a report of objective laboratory indexes in treating DFU with TCMIs such as nerve conduction velocity and hemorheology indexes.

## 5. Conclusion

In management of DF, TCMIs can increase the clinical effective rate of conventional therapies by 27%. Along with a better performance in safety and financial burden, the management of DF can be improved by TCMIs. However, the overall methodological and reporting quality of the included studies was limited. Moreover, there are some contradictions in secondary indexes. Therefore, more high-quality large sample-size RCTs are needed to prove and explain it.

## Figures and Tables

**Table 1:** Basic characteristics of the included studies.

**Study IDs**	**Sample size**	**Age**	**Intervention**		**Duration (weeks)**	**Outcomes**
	**(T/C)**	**(T/C)**	**T**	**C**		
**Chen 2018**	30/30	66.06 ± 0.47(62~82)/ 67.36 ± 0.55(64~84)	Ginkgo biloba extract injection (20ml/d); Basic care	Basic care	4	(1) Total effective rates; (2) Hemorheology

**Chi 2012**	41/41	NA	Erigeron Breviscapus extract injection (30ml/d); Basic care	Basic care	2	(1) Total effective rates; (2) Nerve conduction velocity; (3) Hemorheology

**Guo et al. 2017**	100/100	43.1±3.4/44.3±3.7	Ginkgo biloba extract injection (20ml/d); Alprostadil (10*μ*g/d); Basic care	Alprostadil (10*μ*g/d); Basic care	4	(1) Total effective rates;

**Nuerzada et al. 2018**	31/31	72.5±9.5(63~82)/72.5±10.5(61~83)	Ginkgo biloba extract injection (10ml/d); Basic care	Basic care	NA	(1) Total effective rates; (2) Hemorheology

**He et al. 2010**	40/38	68±14.5/69±15.2	Erigeron Breviscapus extract injection (30ml/d); Basic care	Basic care	2	(1) Total effective rates; (2) Nerve conduction velocity; (3) Hemorheology

**Jin et al. 2017**	70/70	62.79±5.43(53~78)/ 61.97±6.25(52~79)	Compound Salvia Miltiorrhiza injection (20ml/d); Alprostadil (10*μ*g/d); Basic care	Alprostadil (10*μ*g/d); Basic care	4	(1) Total effective rates; (2) Blood lipid levels; (3) Renal function and urine protein

**Lin 2008**	36/36	61.36(51~75)/ 60.35±5.3(49~72)	Erigeron Breviscapus extract injection (400mg/d); Basic care	Basic care	2	(1) Total effective rates; (2) Hemorheology; (3) Ulcer size

**Liu 2008**	37/33	67(55~81)/ 65(56~79)	Danhong injection (NA); Anisodamine (20mg/d); Basic care	Anisodamine (20mg/d); Basic care	4	(1) Total effective rates

**Shi 2010**	42/42	(43~74)/ (38~66)	Compound Salvia Miltiorrhiza injection (12ml/d); Ozagrel (160mg/d); Basic care	Ozagrel (160mg/d); Basic care	4	(1) Total effective rates; (2) Nerve conduction velocity; (3) Hemorheology

**Wang 2010**	22/18	57.6(38~73)/ 56.2(41~71)	Danhong injection (20ml/d); Basic care	Basic care	4	(1) Total effective rates; (2) Hemorheology

**Wen 2014**	20/20	61.8(38~76)/ 63.8(36~75)	Panax notoginsenosides injection (250mg/d); Basic care	Basic care	NA	(1) Total effective rates; (2) Lower limbs blood

**Wu et al. 2003**	36/36	63(48~72)/ 62(46~68)	Compound Salvia Miltiorrhiza injection (10~30g/d); Pancreatic kininogenase (480~720IU/d); Mecobalamin (500~1000*μ*g/d); Basic care	Pancreatic kininogenase (480~720IU/d); Mecobalamin (500~1000*μ*g/d); Basic care	4	(1) Total effective rates; (2) Nerve conduction velocity; (3) Hemorheology

**Xiang 2017**	50/50	58.91±8.54/ 59.42±7.89	Danhong injection (40ml/d); Basic care	Basic care	4	(1) Total effective rates; (2) Ulcer area (3) Arterial diameter and Blood velocity

**Yu et al. 2008**	25/25	57.6(38~73)/ 56.2(41~71)	Danhong injection (40ml/d); Basic care	Basic care	3	(1) Total effective rates

**Zhang et al. 2008**	16/16	52.3±2.5/ 51.8±2.7	Danhong injection (20ml/d); Basic care	Basic care	4	(1) Total effective rates; (2) Nerve conduction velocity

**Zheng et al. 2014**	33/33	58.2±5.3/ 56.7±6.6	Danhong injection (30ml/d); Basic care	Basic care	4	(1) Total effective rates; (2) Oxidative stress status

**Zhu 2010**	30/16	NA	Panax notoginsenosides injection (450mg/d); Basic care	Basic care	1.7(10d)	(1) Total effective rates

**Table 2:** Course of disease.

**Study IDs**	**Women (**%**)**	**Course of Diabetes Mellitus (yrs.)**		**Course of Diabetic Foot**	
		Treatment group	Control group	Treatment group	Control group
**Chen 2018**	45%	9.19±0.28 (5~15)	9.21±0.33 (6~15)	38.24±1.09 (32~78) days	37.94±0.98 (30~76) days
**Chi 2012**	NA	NA	NA	NA	NA
**Guo 2017**	41%	9.6±2.9	9.2±3.5	NA	NA
**Nuerzada 2018**	47%	(5~14)	(5~15)	NA	NA
**He 2010**	46%	15±4.2	16±4.5	5.7±1.5 yrs.	6.8±1.8 yrs.
**Jin 2017**	46%	15.92±4.37 (8~20)	16.35±4.42 (8~21)	1.29±0.34 (0.08~3) yrs.	1.31±0.26 (0.08~3) yrs.
**Lin 2008**	46%	11.2 (5~20)	10.56 (6~18)	15.5 (6~66) mos.	18 (4~72) mos.
**Liu 2008**	46%	11.2 (5~32)		NA	NA
**Shi 2010**	50%	(5~20)	(2~25)	NA	NA
**Wang 2010**	50%	11.7 (4~19)	12.4 (3~21)	13 (3~31) mos.	13.8 (2.5~30) mos.
**Wen 2014**	22.5%	12.4 (2~20)	13.4 (3~21)	NA	NA
**Wu 2003**	50%	7.6 (2~20)	7.8 (1.5~22)	NA	NA
**Xiang 2017**	42%	NA	NA	7.8 ± 2.3 mos.	6.9 ±2.4 mos.
**Yu 2008**	40%	11.7 (4~19)	12.4 (3~21)	13 (3~31) mos.	13.8 (2.5~30) mos.
**Zhang 2008**	40%	5.4±0.3	5.39±0.27	10.1±0.8 mos.	9.5±1.0 mos.
**Zheng 2014**	42.4%	NA	NA	27.0 ±3. 9 mos.	29. 2 ±4.0 mos.
**Zhu 2010**	65%	(5~25)		NA	NA
